# Associations between media use and health information-seeking behavior on vaccinations in South Korea

**DOI:** 10.1186/s12889-017-4721-x

**Published:** 2017-09-11

**Authors:** Jiyeon Kim, Minsoo Jung

**Affiliations:** 1 Korea Centers for Disease Control and Prevention, Cheongju, South Korea; 2 Department of Health Science, Dongduk Women’s University, Seoul, South Korea

**Keywords:** Vaccination, Seasonal flu, Media use, Public health emergency preparedness, South Korea

## Abstract

**Background:**

Although vaccinations are critical for preventing emerging infectious diseases, scant research has been conducted on risk communication. With socio-economic characteristics, health behavior, and underlying diseases under control, we investigated associations between media use, health information-seeking behavior, health information type, and vaccination in the population.

**Methods:**

This study relied on a national survey of Korean adults (*n* = 1367). Participants were adult males and females age 20 and older. Web and face-to-face surveys were conducted throughout July 2014. The main outcome was vaccination (categorized as yes or no). Independent variables were time spent on media, frequency of health information-seeking behavior, and types of health information sought.

**Results:**

Controlling for co-variates, logistic regression analysis was conducted to identify factors that influence Korean adults being vaccinated. Results revealed that accessible information about emerging infectious diseases, listening to the radio, and reading the newspaper were associated with increased odds of being vaccinated. Active seeking health information as well as being female and of higher socio-economic status were positively correlated with Korean adults being vaccinated.

**Conclusion:**

It is critical to promote health information-seeking behavior and use diverse media channels to increase acceptance and awareness of emerging infectious diseases and vaccinations. Because there are differences in vaccination awareness depending on social class, it is critical to reduce communication inequality, strengthen accessibility to vaccinations, and devise appropriate risk communication strategies that ensure Korean adults receive vaccinations.

## Background

In 2015, there were 186 confirmed cases of Middle East Respiratory Syndrome (MERS) in Korea, and 38 deaths from MERS [1]. An investigation analyzed main causes of a failure to prevent these deaths after the MERS outbreak. One of the causes was lack of a national system or effective coordination between experts in managing risk communication at that time [2]. MERS prevention measures were not transmitted in a timely manner through official channels. Instead, inaccurate health-related information proliferated, leading to disease phobia and hysteria among the population [2]. In the case of avian influenza in 2003, media coverage focused overwhelmingly on one aspect of the disease. For this reason, analysis of the virus, infectious pathways, the extent to which the disease spread, preventive measures that could have been taken, and safety issues were relatively neglected [3]. Media plays a critical role in the formation of the population’s perception of social issues [4]. When a new infectious disease emerges, an unfamiliar population perceives that disease according to media reports. Uncertainty and anxiety surrounding the issue are often high [5, 6]. However, this is not the first experience of such a phenomenon amongst Koreans. This is a re-enactment of the disease phobia that transpired in 2008 during the mad cow disease crisis. The recurrence reveals that, despite governmental efforts, Public Health Emergency Preparedness (PHEP) and risk communication strategies have not been sufficiently established or improved in Korea.

An increase in the rise of new infectious epidemics due to globalization highlights the significance of early detection and early response to infections through PHEP. Creating a constant response system is critical. Vaccination is the most effective method of preventing the spread of infectious diseases [7]. Although initially considered a method of disease prevention for infants and toddlers, vaccination is considered critical for adults as well, especially as the number of patients with chronic diseases is increasing in today’s aging society [8, 9]. Now that it is possible to travel around the globe in a day, vaccination against infectious diseases overseas is becoming even more critical.

Vaccination not only protects an individual from an infectious disease, but also protects a local community from that disease from the effect of herd immunity [10]. Health communication strategies and campaigns are imperative in promoting health of the population and strengthening the capacity to effectively respond to diseases [11]. Health experts are placing increasing emphasis on the critical role of risk communication in responding to excessive media coverage and the population’s anxiety [12]. It is critical to enhance the population’s perception of vaccinations by providing accurate information, enabling the population to dismiss inaccurate information on side-effects of vaccinations, and enhance the population’s access to accurate information about vaccinations [13].

The outbreak of MERS has ended. However, similar diseases, such as H1N1, can spread at any time. Preparing non-pharmacological intervention for the population and increasing the vaccination rate are as critical as having a preventive system in place for such situations. Risk communication strategies suitable for socio-contextual conditions must be developed. This study analyzed associations between media use and searching for health information related to vaccinations. Although Korea has a well-established national vaccination program, the 2013 vaccination rate for influenza refers to the number of people aged 65 remains at 21.7% in 2013, that is much lower than the vaccination rate of all OECD countries, 45.87% [14]. Individual and social factors that affect the vaccination rate must be identified.

Previous studies revealed that the vaccination rate amongst individuals suffering from chronic diseases is higher than those without chronic diseases [15, 16]. The more a group practices risky health behavior, such as drinking and smoking, the lower the vaccination rate [17]. Some studies have identified a specific age bracket of those most likely to vaccinate [16–18]. In this age of new media, there is an increasing information gap between people actively seeking information and those not seeking information. This gap impacts health inequalities [19]. Those with lower socio-economic status in particular are more likely to suffer from such a gap [20].

**Fig. 1:**
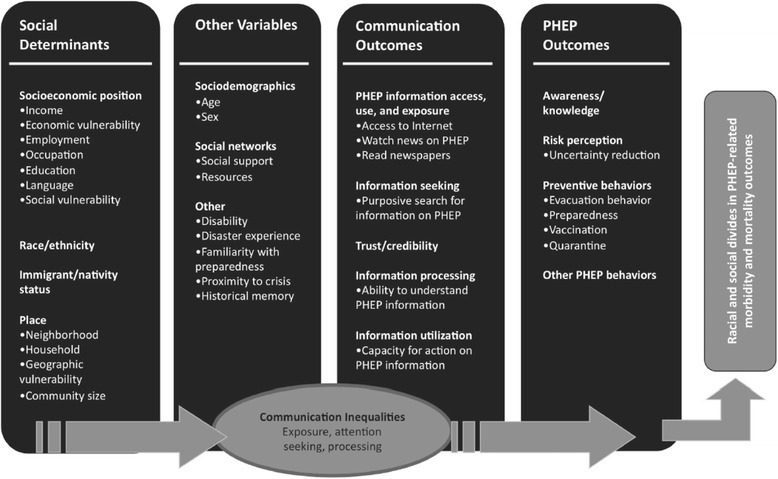
Structural influence model of communication inequalities [8]

Seeking health information has become more critical than in the past as epidemiological characteristics of the most prevalent diseases have changed from infectious to chronic diseases. This study identified the association between media use and seeking health information about vaccinations. As discussed above, various factors may affect vaccination rates. These factors impact communication inequality. Communication inequality refers to the theory that a gap in accessibility to information and the ability to process that information exists between social classes [21]. As shown in Fig. 1, it defined as the difference in exposure to public health communication messages and the capacity to access, process, and act upon information, are influenced by social determinants, and may result in significant disparities in health-related knowledge, behavior choices, and ultimately, health outcomes [8]. This study was conducted to reduce communication inequality and identify appropriate risk communication strategies that may increase the vaccination rate.

## Methods

### Respondents

This study used primary data obtained from a national survey funded by the government. The data used for this study is from a survey of 1367 respondents from a nationally representative sample of Korean adults that participated in the Hankook Research Master Sampler Panel^©^. The Master Sampler consists of 350,000 statistical data points from a selection of individuals whose various regions, gender, ages, jobs, academic background, and income distribution are representative of the larger population. Members of this panel were recruited using a dual sampling frame, a combination of Random Digit Dial and Address-Based Sampling for sampling of individuals without telephone land lines. We used face-to-face surveys and computer-aided web surveys mixed method research. For face-to-face surveys, 501 adults living in Seoul, Gyeonggi, and Incheon regions of Korea were surveyed; for computer aided web surveys, 1010 adults living in 16 metropolitan cities in Korea were surveyed. In web surveys, panels of respondents were statistically representative of Korea’s population in terms of region, gender, age, occupation, education, and income distribution. Mixed method surveys were used to increase sample representation by approaching sample households in a variety of ways. Participants received nominal cash incentive to participate in the survey. Final response rate was 87.0%. Survey questions with missing values for key analytical variables were excluded using a pairwise method.

### Measures

This work was supported by a grant from the National Research Foundation of Korea funded by the Korean government. All datasets and survey questionnaires are available at (https://www.nrf.re.kr/cms/page/main?menu_no=41). The survey questions were from previous reports on the effect of social context (including mass media use and social capital) on health [8, 13, 20]. Questionnaires addressed topics adapted from the Health Information National Trends Survey (http://hints.cancer.gov/).

#### Dependent variable

The main outcome variable was vaccination for oneself (‘*Now, think about getting the seasonal flu vaccine for yourself. Have you vaccinated over the past one year?’*). Responses were grouped in two categories: yes or no.

#### Independent variables

Media use: General mass media usage was assessed using the following questions: “In the past 7 days, how many hours did you watch television per day on average? Listen to the radio? Read a newspaper? Search for information with a smartphone? Read news on the Internet with a personal computer?” Possible responses were “30 min or less,” “30 min to 1 h,” “1 to 2 h,” “2 to 3 h,” or “3 h or more.”

Health-related Information-Seeking Behavior (HISB): Respondents were asked to rate their health-related information-seeking activity on a five-point Likert scale ranging from very active to very inactive with the following questioning: “Thinking about all the times you have looked for health-related information from any source, how much do you search for information about health?” Responses were grouped in the following five categories: “very inactively,” “somewhat inactively,” “average,” “somewhat actively,” or “very actively.” Types of health-related information were assessed with the following questioning: “In the past one month, have you ever searched for information on diseases you have? General health-related information? Information on hospitals and doctors? Information on how to quit smoking or drinking alcohol?” The responses were grouped into two categories: yes or no.

#### Covariates

Participants’ socio-economic position was measured by educational attainment and annual household income. For income, respondents were asked about total annual household income before taxes, divided into the following categories: under U.S. $20,000; $20,000-39,999; $40,000-59,999; $60,000-$79,999; and $80,000 or more. For education, respondents were asked to identify the highest level of education completed (high school diploma or less, college degree, or postgraduate degree). Three health behavior variables indicating smoking and drinking alcohol were used in regression analyses. A value of 0 was assigned to “never smoked” for smoking and “did not drink during the past month” for drinking alcohol. All other responses were considered to indicate smokers and drinkers and were assigned a value of 1. Other covariates were age, gender, and presence of underlying diseases.

### Statistical analyses

Descriptive statistics and frequencies were derived for socio-economic characteristics of study subjects. Bivariate analyses were undertaken for each potential predictor variable to identify socio-contextual factors associated with vaccinations. Differences in general characteristics between vaccination and non-vaccination groups were determined using chi-squared tests. Multivariate logistic regression analyses were used to analyze potentially influential factors related to seasonal flu vaccination of adult Korean males. The effect size of regression models, that is the ratio of variance, is presented in this study as the Nagelkerke value, that is pseudo-R.^2^ All statistical analyses were conducted using STATA v.12.0 (STATA, College Station, TX).

### Ethics statement

Approval for this study was granted by the Korea National Institute for Bioethics Policy Institutional Review Board (April 11, 2014; P01-201404-SB-19-00). Subjects provided written informed consent to participate in this study. The Ethics Committee of the Demographic Health Survey approved the consent procedure. No information that can publicly identify individual participants was collected during the data collection process.

## Results

### General sample characteristics

Of 1367 participants, 49.0% are women and 51.0% are men (Table 1). Regarding ages, 20.0% are in their 40s while 20.1% are age 60 or older. Approximately 33.9% of participants earn $20,000-$40,000 USD per year. Most (62.3%) participants have a college degree or higher (Table 1).

**Table 1:** General characteristics of the sample (*n* = 1367)

Category		%	n
Gender	Male	51.0	697
	Female	49.0	670
Age	20-29	20.3	278
	30-39	19.6	268
	40-49	20.0	274
	50-59	19.9	272
	60-69	20.1	275
Education	High school diploma or less	37.8	516
	College	50.7	693
	Post-graduate degree	11.6	158
Annual Income	Less than US$20,000	16.3	223
	US$20,000-40,000	33.9	464
	US$40,000-60,000	28.2	386
	US$60,000-80,000	12.9	177
	US$80,000 or more	8.6	117

### Differences between the vaccinated group and non-vaccinated group

As shown in Table 2, compared to the non-vaccinated group, the vaccinated group had more women (χ^2^ = 20.074, *p* < 0.001), more individuals older than age 60 (χ^2^ = 81.094, *p* < 0.001), and more individuals earning higher annual income (χ^2^ = 10.927, *p* < 0.05). With respect to health behavior and health status, the vaccinated group had more non-smokers (χ^2^ = 7.865, *p* < 0.05) and more individuals with underlying diseases (χ^2^ = 43.742, *p* < 0.001). Regarding media use, the vaccinated group had more individuals exposed to television (χ^2^ = 9.669, *p* < 0.05), radio (χ^2^ = 22.867, *p* < 0.001), and newspapers (χ^2^ = 34.599, *p* < 0.001). There was no statistically significant correlation between Internet searches conducted with smartphones or personal computers and being vaccinated. The vaccinated group was associated with those more actively seeking health-related information (χ^2^ = 53.693, *p* < 0.001). This association was consistent regardless of the type of health-related information sought.

**Table 2:** Differences between the vaccinated group and the non-vaccinated group

Category			Non-vaccinated group (*n* = 859)	Vaccinated Group (*n* = 508)	x^2^ (*p*-value)
Socio-demographic Characteristics	Gender	Male	68.6	31.4	20.074 (0.001)^***^
		Female	56.9	43.1	
	Age	20-39	76.3	23.7	81.094 (0.001)^***^
		30-39	55.2	44.8	
		40-49	63.9	36.1	
		50-59	73.9	26.1	
		60 or older	44.7	55.3	
	Annual Income	Less than US$20,000	65.0	35.0	10.927 (0.027)^*^
		US$20,000-40,000	65.1	34.9	
		US$40,000-60,000	64.0	36.0	
		US$60,000-80,000	60.5	39.5	
		US$80,000 or more	62.8	37.2	
	Education	High school or less	68.8	31.2	15.176 (0.004)^**^
		College	60.6	39.4	
		Post-graduate	60.1	39.9	
Health Behavior & Diseases	Drinking Alcohol	No	59.4	40.6	22.970 (0.001)^***^
		Yes	73.4	25.7	
	Smoking	No	57.0	43.0	7.865 (0.005)^**^
		Yes	65.1	34.9	
	Underlying disease	No	68.1	31.9	43.742 (0.001)^***^
		Yes	48.6	51.4	
Media Use	Television Watching	30 min or less	71.1	28.9	9.669 (0.046)^*^
		30 min to 1 h	68.5	31.5	
		1 to 2 h	59.5	40.5	
		2 to 3 h	63.0	37.0	
		3 h or more	59.8	40.2	
	Radio Listening	30 min or less	66.5	33.5	22.867 (0.001)^***^
		30 min to 1 h	52.9	47.1	
		1 to 2 h	59.8	40.2	
		2 to 3 h	47.1	52.9	
		3 h or more	49.0	51.0	
	Newspaper Reading	30 min or less	66.2	33.8	34.599 (0.001)^***^
		30 min to 1 h	52.0	48.0	
		1 to 2 h	54.5	45.5	
		2 to 3 h	27.8	72.2	
		3 h or more	0.0	100.0	
	Smartphone Browsing	30 min or less	61.5	38.5	n.s.
		30 min to 1 h	63.5	36.5	
		1 to 2 h	68.7	31.3	
		2 to 3 h	57.7	42.3	
		3 h or more	57.8	42.2	
	Computer Searching	30 min or less	61.5	38.5	n.s.
		30 min to 1 h	57.5	42.5	
		1 to 2 h	65.7	34.3	
		2 to 3 h	65.3	34.7	
		3 h or more	66.7	37.2	
Information Search	Health Information Seeking Behaviors	Very inactively	82.0	18.0	53.693 (0.001)^***^
		Somewhat inactively	71.7	28.3	
		Average	66.5	33.5	
		Somewhat actively	52.3	47.7	
		Very actively	42.9	57.1	
Types of Health-related Information	Own diseases	Never searched	72.9	27.1	40.676 (0.001)^***^
		Searched	55.9	44.1	
	General health	Never searched	70.6	29.4	14.453 (0.001)^***^
		Searched	59.7	40.3	
	Hospitals and doctors	Never searched	69.1	30.9	43.153 (0.001)^***^
		Searched	51.1	48.9	
	Renounce smoking/drinking	Never searched	66.8	33.2	7.690 (0.006)^**^
		Searched	59.5	40.5	

*n.s.* not significant

*: *p* < 0.05, **: *p* < 0.01, ***: *p* < 0.001

### Socio-contextual determinants of vaccination for seasonal flu

According to the final model (Table 3), that used logistic regression to analyze socio-contextual determinants of vaccination while controlling for socio-demographic characteristics and health behavior, those with underlying diseases are 69.1% more likely to be vaccinated than those without underlying diseases (OR = 1.691, 95% CI: 1.261-2.267, *p* < 0.001). Regarding media use, those that spent more time listening to the radio are 13.9% more likely to be vaccinated than those that did not listen to the radio (OR = 1.139, 95% CI: 1.016-1.277, *p* < 0.05). Those that spent more time reading print newspapers are 31.9% more likely to be vaccinated than those that do not read print newspapers (OR = 1.319, 95% CI: 1.100-1.581, *p* < 0.01). However, those that spent more time using the Internet are 17.7% less likely to be vaccinated (OR = 0.823, 95% CI: 0.743-0.911, *p* < 0.001). Those that more actively engaged in health information seeking are 22.7% more likely to be vaccinated (OR = 1.227, 95% CI: 1.048-1.436, *p* < 0.05). Regarding contents of health-related information that participants sought, the more the topic was about diseases suffered by information-seekers or hospitals and physicians, the more likely these individuals will be vaccinated.

**Table 3:** Associations of media use, health information seeking behaviors, and seasonal flu vaccination

Category		Model 1		Model 2		Model 3		Model 4	
		OR	95% CI	OR	95% CI	OR	95% CI	OR	95% CI
Socio-demographic Characteristics	Gender(Ref.: Male)	1.692^***^	1.349-2.121	1.556^**^	1.202-2.015	1.619^***^	1.235-2.124	1.525^**^	1.157-2.009
	Age	1.229^***^	1.133-1.332	1.103^*^	1.007-1.207	1.106	0.994-1.230	1.095	0.983-1.220
	Education	1.141	0.947-1.335	1.109	0.945-1.301	1.114	0.943-1.316	1.034	0.870-1.229
	Annual Income	1.121^*^	1.015-1.239	1.121^*^	1.013-1.240	1.114	0.996-1.224	1.118^*^	1.006-1.241
Health Behavior & Diseases	Drinking Alcohol (Ref.: None)			0.636^**^	0.463-0.872	0.642^**^	0.465-0.886	0.656	0.472-0.911
	Smoking (Ref.: None)			0.964	0.739-1.256	0.917	0.700-1.201	0.894	0.679-1.177
	Underlying Diseases			2.124^***^	1.618-2.787	2.122^***^	1.609-2.798	1.691^***^	1.261-2.267
Media Use	Television Watching					0.969	0.875-1.072	0.963	0.868-1.068
	Radio Listening					1.176^**^	1.052-1.314	1.139^*^	1.016-1.277
	Newspaper Reading					1.385^***^	1.159-1.655	1.319^**^	1.100-1.581
	Smartphone Browsing					1.154	1.032-1.289	1.116	0.996-1.249
	Computer Searching					0.868^**^	0.787-0.957	0.823^***^	0.743-0.911
Health Communication	Health Info. Seeking Behavior							1.227^*^	1.048-1.436
	Own diseases							1.460^*^	1.097-1.944
	General health							0.905	0.664-1.234
	Hospitals and doctors							1.548^***^	1.185-2.023
	Renounce smoking/drinking							1.050	0.802-1.374
-2LL		1750.494		1712.467		1674.987		1632.365	
Nagelkerke R^2^		0.052		0.088		0.123		0.161	

*: *p* < 0.05, **: *p* < 0.01, ***: *p* < 0.001

## Discussion

With widespread danger of new infectious diseases, increasing the population’s vaccination rate is critical for public health. This study was conducted to identify socio-contextual factors that promote preventative behavior regarding vaccinations from the perspective of health communication. Results of this study revealed those that actively engaged in HISB were more likely to be vaccinated than those that did not actively engage in HISB. This matches results of earlier studies revealing an association between media use, HISB and vaccination rates [8]. Media use promotes healthy practices as it provides a means of communicating and explaining healthcare-related knowledge to the population [22]. The population is positively or negatively influenced by media in addition to medical staff and family members relative to critical behavioral decisions such as being vaccinated. For example, pregnant women using the Internet are influenced by other pregnant women’s positive experiences with vaccinations [23]. Health information seeking behavior and use of media significantly influence healthy lifestyles, early diagnosis, and sensible disease management [24]. Meanwhile, vaccination rates were higher with female respondents that have high income levels, or underlying diseases. Those suffering from chronic diseases were more attuned to the significance of disease prevention. Respondents sought disease-related information more often than those that are healthy. Such tendencies regarding information seeking promoted disease prevention behavior such as being vaccinated, consistent with results of previous studies [15, 17]. However, those with lower socio-economic or health status had less accessibility to information or capacity to use that information, that is in agreement with results of previous studies [19, 20]. Consequently, it is imperative to increase health equity in vaccination rates by mitigating communication inequalities.

Health communication variables such as media use and HISB may affect various types of health-related preventative behavior. For example, disease prevention behavior including vaccination may be promoted if information accessibility is heightened and an individual’s health information-seeking capacity is strengthened in populations with serious health inequality, especially for those with low income [16, 25]. Previous studies have mainly focused on determining risk factors for health inequality. Studies on protective factors have been insufficient. This study reveals how health communication variables are linked to vaccination status. Consequently, it is critical to consider policies that will reduce health disparities stemming from communication inequality by providing useful information on new infectious diseases or vaccination in an easily accessible manner to vulnerable groups often excluded from valuable health-related information. From the perspective of health communication, it cannot be stated with certainty that the use of media or exposure to specific health information will lead to respondents’ behavior only in a positive direction (e.g., vaccination uptake). This is because while content of news is thoroughly verified for network television, information on the Internet includes inaccurate and misleading news and anti-vaccine information as well. Such organizations create closed or secret websites and exchange information among themselves to avoid social controversy. Our study used searches only on Internet news, excluding social networking services (SNS) and blogs to preserve integrity and reliability. To understand how specific health information through certain media is distributed about people’s health behavior, content analysis and small-scale qualitative research are critical. Before exploring this stage, our study sought to understand vaccination behavior from the viewpoint of population-based behavioral medicine.

Future studies should explain how diverse communication choices affect being vaccinated. In a small country with outstanding media environment including South Korea, it is difficult to understand ultimate health results from the selection of communication channels. This is because most individuals simultaneously watch diverse media channels and receive large volumes of information. Consequently, this study focused on desirable health behavior (e.g., vaccine uptake) and analyzed characteristics of groups that did not adopt such behavior. Vaccination compliance was generally low among groups with low socio-economic status, and we hypothesized that the issue of an information gap mediated between the two factors. This study was conducted to validate the existence of communication inequality as the strong link between social inequality and health inequality and to identify ways of increasing health equity through the mediating effect of communication inequality.

This study had several limitations. First, because it used cross-sectional data, the possibility of reverse causality cannot be dismissed. Consequently, caution must be exercised in establishing causal relationships among respondents’ vaccination status, media use, and HISB. Findings must be supplemented with longitudinal data. Second, this study did not ask specifically from which types of media respondents acquired health-related information and what type of health-related information was acquired. However, it is difficult to acquire such information from a study design and combine them in a model. Third, vaccination rate was highest among those age 60 and older. In Korea, the elderly (older than age 65) are vaccinated at no cost annually. The possibility that no-cost vaccines contribute to the vaccination rate cannot be dismissed. However, in previous studies that excluded subjects receiving no-cost vaccinations, the older the age, the higher the vaccination rate. As age progresses, the need for vaccination increases and the vaccination rate also increases. Fourth, the explanatory power of this model is somewhat low (16.1%) because other complex factors additionally influenced respondents’ seasonal flu vaccination, that was the dependent variable. In social science, the explained variance of regression models is judged to be outstanding if it totals 20-30%. However, this study constructed the optimal model from the perspective of behavioral medicine and results of regression diagnosis did not exhibit non-systematic residuals separately.

## Conclusion

The more a society’s media environment evolves through information and communication technology, the more quality knowledge circulates in a virtuous circle. The greater communication inequality is, the more likely health inequality will worsen. Reducing the information gap among social classes through a healthy media environment is a way of achieving health equity. Findings of this study will enable development of PHEP strategies by mitigating communication inequality. This study has practical implications for implementation of vaccination campaigns that use effective, timely health communication. To increase the population’s vaccination rate, it is critical to ensure appropriate use of radio and print newspapers. Health-related information should be provided to patients by public health personnel at medical agencies in a more accurate and accessible manner. It is imperative that responsible media representatives strengthen the population’s health information-seeking access through viable, diverse media channels. As the population’s growing fear of epidemics has become a common phenomenon, elucidating social determinants of preventative behavior such as vaccination is a critical task for public health workers. Consequently, multilevel risk communication strategies must be developed to increase the population’s vaccination rate.

## Abbreviations

HISB: Health-related Information-Seeking Behaviors
MERS: Middle East Respiratory Syndrome
OECD: Organization for Economic Co-operation and Development
PHEP: Public Health Emergency Preparedness
